# Is chubby cute? Clinical perspectives on obesity prevention and metabolic health in Macau

**DOI:** 10.3389/fpubh.2026.1759597

**Published:** 2026-02-11

**Authors:** Wengioi Mio, Caiyi Tan, Puikei Tou, Sipui Cheang, Chonin Cheang

**Affiliations:** 1 Macau Yin Kui Hospital, Macau, Macao SAR, China; 2 Macau Society for Health Economics, Macau, Macao SAR, China

**Keywords:** clinical practice, Guangdong-Hong Kong-Macau Greater Bay Area, Macau, obesity, public health

The traditional cultural perception of “chubby” infants as symbols of health and vitality has inadvertently contributed to widespread nutritional excess among younger populations in Macau, precipitating an alarming rise in obesity-related metabolic disorders (1). Contemporary epidemiological data underscore the urgency: over half of Macau's adult population (51.9% in 2016) exhibits overweight or obesity, representing a 5.7% increase from 2006 (1); among secondary school students, approximately 21.7% are overweight or obese (2). These statistics transcend academic interest—they reflect the daily clinical reality facing Macau's healthcare providers.

When the Macau Health Bureau articulated the goal of “curbing the obesity rate rise by 2030” (3), healthcare professionals recognized that this mandate extends beyond the clinic. The obesity crisis demands simultaneous intervention across medical systems, policy frameworks, and community environments. We propose a three-pronged clinical action strategy addressing this multifaceted challenge.

## System-level barriers and proposed solutions

Macau's healthcare infrastructure, while incorporating progressive elements such as tiered diagnostic frameworks and digital health platforms (e.g., “My Health 2.0”) (4), faces critical bottlenecks. First, referral pathways remain inefficient, creating prolonged treatment delays that allow patients to experience disease progression and preventable complications. Second, a shortage of specialized professionals—particularly registered dietitians and metabolic health specialists—constrains capacity. Third, limited drug accessibility and public awareness of metabolic disease manifestations (exemplified by delayed recognition of acanthosis nigricans as an insulin resistance marker) perpetuate diagnostic delays (5, 6). Notably, in our clinical experience, we have observed acanthosis nigricans in patients as young as 6 years old, indicative of early-onset insulin resistance and substantially elevated future risk of type 2 diabetes and other metabolic syndrome components (5, 6). Fourth, external environmental factors, including food delivery platforms that algorithmically promote calorie-dense options and generic physical activity messaging, paradoxically undermine targeted intervention effectiveness.

To address these barriers, we advocate for: (1) establishing expedited referral pathways for high-risk patients (BMI >35 or those with multiple metabolic comorbidities), with a proposed target assessment intervals of ≤ 14 days, based on clinical consensus for timely intervention; (2) rapidly expanding the professional workforce through cross-border credential recognition and public-private partnerships, drawing inspiration from international programs such as the UK's NHS Weight Management Partners scheme (7), while acknowledging the need for adaptation to Macau's specific context; (3) exploring regulatory measures that could require food delivery platform algorithms to display nutritional indices (including glycemic index values) and preset healthy default options; and (4) implementing pilots of age-stratified metabolic screening programs at critical developmental windows (e.g., males aged 8–9 years, females aged 10–11 years, when BMI growth velocity peaks).

## Reframing treatment paradigm: from weight to metabolic health

A fundamental limitation of current obesity management lies in over-reliance on Body Mass Index (BMI) as the primary outcome metric. Evidence increasingly demonstrates that individuals with normal BMI can harbor significant visceral adiposity and insulin resistance, conferring substantial cardiovascular risk. We propose shifting the therapeutic paradigm toward metabolic health indicators, specifically visceral fat area (VFA ≥90 cm^2^ represents a critical threshold for cardiovascular complications in type 2 diabetes patients) (8). Weight reduction of 5 kg, when accompanied by preserved or increased muscle mass and improved insulin sensitivity (reflected by a target HbA1c reduction of 2.3% via low-glycemic-load dietary intervention), generates superior clinical outcomes compared to equivalent weight reduction with muscle loss (9, 10).

This paradigm shift necessitates: (1) equipping community health centers with bioelectrical impedance analysis devices to quantify body composition changes beyond weight reduction, enabling detection of unfavorable body composition shifts (e.g., 3 kg weight loss accompanied by 1.5 kg muscle loss) (10); (2) prioritizing glycemic control outcomes (e.g., HbA1c reductions of 2.3% via low-glycemic-load dietary intervention) as therapeutic endpoints (9); and (3) adopting strength-training interventions for high-risk populations with mobility constraints, addressing metabolic dysfunction while mitigating fall risk.

However, the widespread implementation of BIA and specialized screening requires consideration of feasibility, including costs, workforce training, and potential risks of over-medicalization. A phased approach, starting with high-risk groups and leveraging public-private partnerships, could be a pragmatic first step.

## Policy advocacy as clinical responsibility

Obesity prevention transcends individual behavioral modification—it demands environmental restructuring. We recommend considering legislative action mandating food health labeling (incorporating glycemic index values), implementing taxation on sugar-sweetened beverages in schools [Evidence from settings like Mexico shows potential for consumption reduction, particularly among low-income populations (11, 12)], and establishing standardized nutrition criteria for institutional food services. Current initiatives such as the “Healthy Eateries” program, with over 160 participating establishments, have demonstrated commercial viability—according to program reports, participating merchants reported 20% annual business growth (13), providing evidence that public health objectives and commercial success can align.

Critically, healthcare professionals must evolve beyond traditional diagnostic roles. Engaging in policy advocacy, collaborating with urban planners on walkable infrastructure development (exemplified by Macau's Black Sand Bay Waterfront 350-meter fitness corridor), and partnering with commercial sectors on “default option” restructuring represent emerging and essential extensions of clinical functions. We posit that the pediatrician diagnosing acanthosis nigricans in a 6-year-old child has a role to play in addressing the food environment that generated the insulin resistance, as part of a comprehensive approach to patient care.

## Leveraging data and innovation

Macau's existing digital health infrastructure (My Health 2.0 platform) (4) offers unprecedented opportunities for population risk stratification. By integrating BMI, metabolic parameters, and behavioral data, primary care providers can identify high-risk individuals for targeted intervention earlier in disease trajectories. Furthermore, preliminary data from the “Healthy Eateries” program demonstrate that commercial entities and public health objectives need not be antagonistic (13).

The implementation of color-coded beverage labeling systems in schools (as exemplified by red-yellow-green classifications at HoiKong Middle School) creates decision environments requiring minimal willpower, significantly reducing sugar-sweetened beverage consumption among adolescents (7). Such environmental restructuring strategies prove particularly effective when compared to traditional public health messaging alone.

## Conclusion

Achieving the 2030 obesity stabilization target demands that clinicians reconceptualize their professional scope (3). Individual consultations and prescription writing remain essential but insufficient. Healthcare professionals must simultaneously function as policy advocates, environmental architects, and population health stewards. The promise of a “Healthy Macau” will be realized only when clinical expertise informs not merely treatment protocols, but legislative frameworks, urban design, and commercial algorithms. In this paradigm, preventing the initiation of insulin therapy ranks equal to its prescription, and ensuring that students encounter vegetable vendors instead of beverage kiosks en route home becomes as clinically relevant as any pharmaceutical intervention.
